# Steady state analysis of modern industrial variable speed drive systems using controllers adjusted via grey wolf algorithm & particle swarm optimization

**DOI:** 10.1016/j.heliyon.2020.e05438

**Published:** 2020-11-08

**Authors:** Safwan Nadweh, Ola Khaddam, Ghassan Hayeh, Bassan Atieh, Hassan Haes Alhelou

**Affiliations:** a Electrical Power Engineering Department, Tishreen University, 2230 Lattakia, Syria; b Mechatronics Engineering, Manara University, Lattakia, Syria

**Keywords:** Electrical engineering, Energy, Industrial engineering, Electrical system, Four quadrant chopper, Variable speed drive system, Power quality, Grey wolf optimization, Particle swarm optimization

## Abstract

This paper presents the reconfiguration of control circuit designed to control four-quadrant chopper placed in the variable speed drive system (VSDS)'s DC-link. The purpose of this design is to reduce the overall total harmonic distortion THD% of input current, and the ripple factor (RF) of the DC-link current in this system. Both of Grey Wolf Algorithm (GWO) & Particle Swarm Optimization (PSO) have been used to get the optimal parameters of proportional integral PI and proportional integral differential with filter PIDN controllers. The variable speed drive system and the proposed filter have been modeled in integration with the suggested algorithms to determine the optimal values of the controllers' parameters. The grey wolf algorithm GWO outperformed the PSO algorithm in term of reaching the optimum parameters in less number of iterations in both dynamic and static work conditions. Also, the time response of the system with GWO is better than with PSO.

## 1 Introduction

The widespread of high-performance variable speed drive systems in modern industrial applications imposes a necessity to develop the control circuits of these systems in order to follow drive commands quickly and accurately in accordance with load requirements. Due to the fact that VSD systems have a significant impact on the power quality, it is important to improve the efficiency of control circuits in these systems [1]. The speed regulation of electrical motors used in both DC and AC electrical drive systems suffers many problems, the most important of which are system parameters selection, controllers' parameters tuning. When designing any drive system, it is necessary to monitor changes in network parameters in different system types. In addition, a comprehensive study of the effects of low power quality indices must be conducted. Various scientific organizations have set power quality standards and indicators such as IEEE-519, IEC, and others [2]. The main objective of these standards is to set limits on network variables that should not be exceeded such as *f,* Δ*u, THD%.*

To achieve power quality specifications in variable speed drive systems, many studies have been conducted using traditional and digital control devices such as PI, PID, PIDN, FLC, neural networks, Fuzzy and Swarm algorithms. The objective of the various control methods is to improve power quality. Recent statistics indicate that more than 95% of industrial control applications have used PID controllers, and these controllers can be tuned in different ways such as; manual tuning method, Ziegler-Nichols method. Many researchers proposed several control methods in order to achieve the objectives of design specifications. In [3] Robak et al. introduced a method for tuning the parameters of PID controller used in the control circuits. The PID parameters have been adjusted independently depending on the step response and the demand response. A good dynamic and static performance have been achieved with the resulted parameters used to regulate the motor speed. In 2017, Sekar et al. [4], proposed a two-level hysteresis control method and it gave acceptable results in terms of quality but caused two levels of operation and two-phases switching characteristics. The study showed that THD % of input currents reached values up to more than 100% when using conventional AC drive systems. When using the proposed control scheme THD% have been reduced to 30%. In 2017, Walcott et al. [5], proposed a three-phase PWM control scheme which has proven to be very effective in reducing the ripples of voltage and current in both DC-link, and grid sides. THD% of grid-side current has been reduced to with 28% using the proposed control. In 2018, Gamit et al. [6], used fuzzy logic instead of traditional controllers to control both DC-link voltage and current by choosing several fuzzy sets and the proposed control outperformed the above-mentioned methods by reducing the THD% value to 16%. In [7] researchers suggested a scheme of a rectifier with five levels that can be controlled by using Sine pulse width modulation SPWM technique in order to achieve a changeable speed of a DC machine with fixed load torque and low harmonic distortion in Ac supply side. The authors in [8] presented a fuzzy logic speed controller for a voltage inverter and asynchronous motor drive system. The switches of the inverter have been controlled using the SPWM modulation technique and results showed that the suggested controller has achieved an adequate speed regulation at high mechanical load changes, nevertheless, it gave small steady error and the controller has been found to be unstable at extremely low speed or high-speed change.

Optimization algorithms such as particle swarm optimization algorithm PSO, genetic algorithm GA, and others have demonstrated high efficiency and good results by improving steady state characteristics [9, 10, 11, 12, 13, 14, 15, 16, 17, 18, 19, 20]. Some of the VSDSs used in modern industrial applications have demonstrated some important features, such as simplicity, easiness, and high reliability of the application. The main task of optimization algorithms is to achieve the optimal design according to a set of international standards to obtain the highest effective solution in terms of cost, performance, productivity, reliability, and efficiency. The grey wolf optimization algorithm (GWO) was used to ensure optimization, simplification, rapid convergence and improved stability of the studied systems (each system according to its mode of operation). The grey wolf optimizer introduced a well-understood method for optimizing the fuzzy controllers and adjusting the motor speed to ensure high performance control in order to reduce the speed wave at low speed task to overcome the speed fluctuation of a low-torque high-speed operating system, as in the case of continuous magnet motors.

This paper introduces a new technology to adjust both PI and PIDN parameters used to generate control signals needed to drive the four-quadrant chopper transistors in the studied system by comparing between GWO & PSO algorithms. The most important objective of this system is to increase the quality of the input current by reducing the THD% factor and RF factor of DC-link current. A comparison between GWO with PSO is conducted in terms of the minimum value of the objective function (integration of the absolute value error), minimum number of iterations and best time response for studied system.

This paper introduces an improved control scheme to enhance the quality specifications of VSDs in industrial facilities. The proposed study has been conducted by modeling high-power VSDS with four-quadrant chopper using Matlab Simulink, choosing the target function, tuning the parameters of the PID and PIDN controllers using both GWO & PSO algorithm, and evaluating the effect of using a GWO and PSO tuning on the performance of the studied system. The literature review is performed in section 1, the methodology of optimization algorithms is clarified in section 2. Section.3 show both of the studied system and control strategy. Results and discussion are shown in section 4, and finally conclusion and recommendations are presented in section 5.

## 2 Optimization algorithms

Optimization algorithms reach the optimal value by increasing or decreasing an objective function (E (x)), which is simply an arithmetic function, based on the internal parameters of the learning model used to calculate the objective value (y) of a set of predictive values (x) used in the model. Traditional optimization techniques help to achieve the optimal solution for continuous and discrete functions. In 2014, Mirjalili et al. [9], proposed the optimization concept includes computation of variants, control theory, theories of optimization, decision theory, games theory, and linear programming, Markov chains, network analysis, optimization theory, queuing systems, etc. It is well known that optimization is the procedure of discovering the best solution for a particular issue to achieve a specific objective by making changes to the initial solution and using the acquired information to reach the optimal solution. In 2010, Kelley et al. [10], defined the optimization model as a mathematical model to improve (reduce or increase) the target function without overriding key constraints, known as mathematical programming. The optimization issue in computer and mathematics science is concerned in finding the finest solution between all probable solutions. The optimization problems can be separated into two groups depending on the nature of the variables (discrete or continuous). With the advent of the computer, optimization becomes part of the computer-aided design activities. The problem of the optimal solution should be formulated for static or dynamic data taking into account the single and multiple objectives [9, 10, 11, 12]. Meta-Heuristic optimization has become very common over the last decades because of its simplicity and flexibility. These techniques are related to natural phenomena (animal behavior), or evolutionary concepts which attracts researchers to develop and propose new methods.

### 2.1 Particle swarm optimization (PSO)

**Figure 1:**
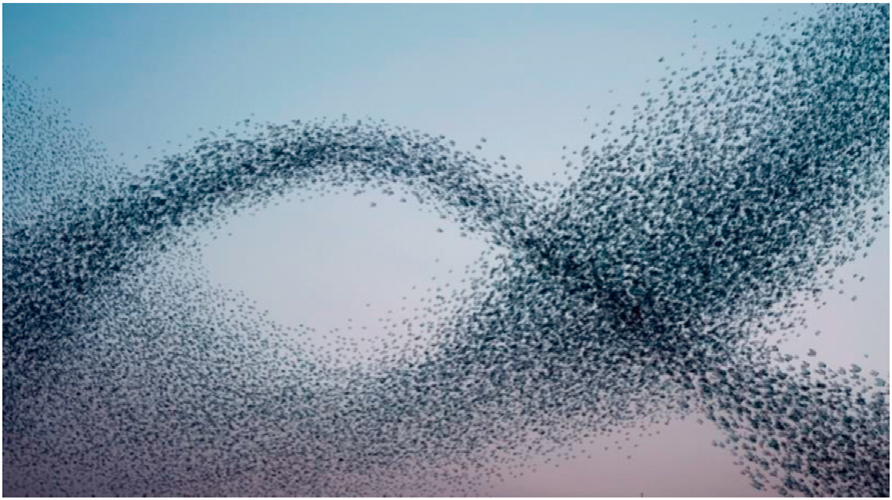
Particle swarm optimization.

In 2007, Kennedy et al. [13], Inspired the particle swarm optimization algorithm (PSO) which is a population-based stochastic algorithm driven by the intelligent common behavior of some animals such as flocks of birds or schools of fish. PSO is a computational technique that finds the optimal solution for a problem by frequently trying to promote a candidate solution. PSO is similar to evolutionary computation techniques such as Genetic Algorithms (GA). The algorithm starts with a population of random solutions (particles) and tries to find the optimal solution by updating generations. In every iteration, each particle is updated by following two “best” values. The first one is the best solution (fitness) that has been achieved so far. Another “best” value that is tracked by the particle swarm optimizer is the best value obtained so far by any particle in the population. Figure 1 shows the particle swarm optimization.

**Figure 2:**
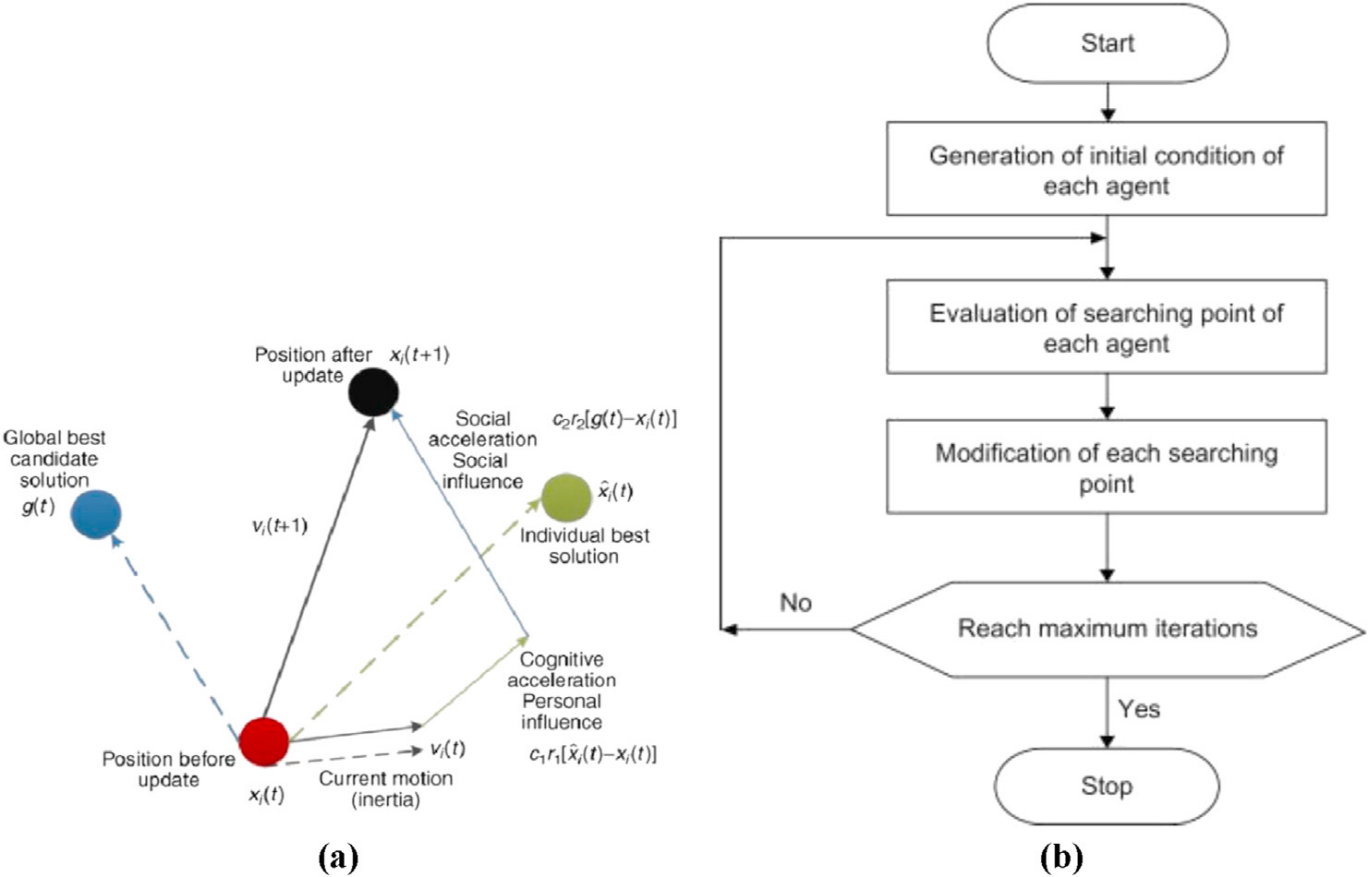
PSO algorithm: (a) Main steps of PSO, and (b) The general flowchart of PSO search algorithm.

PSO recognized originally by Kennedy, Eberhart, and Shi first proposed PSO for emulating social manner as a formal impersonation of the activity of members in a bird flock or fish school. The algorithm has been simplified and observed to perform optimization. PSO algorithm can search very large spaces of candidate solutions. However, Metaheuristics such as PSO do not promise that the best solution will be certainly found. Also, PSO does not use the gradient of the issue being optimized, which means PSO does not require the optimization problem to be differentiable as in traditional optimization methods such as gradient-based and quasi-newton methods. Figure (2a) shows the steps of PSO and Figure (2b) shows the flowchart of the PSO search algorithm [14, 15].

### 2.2 Grey wolf optimization (GWO)

GWO algorithm is an improved version of PSO algorithm and it was proposed in this paper to find the optimal values for the parameters of the controllers used in the study.

**Figure 3:**
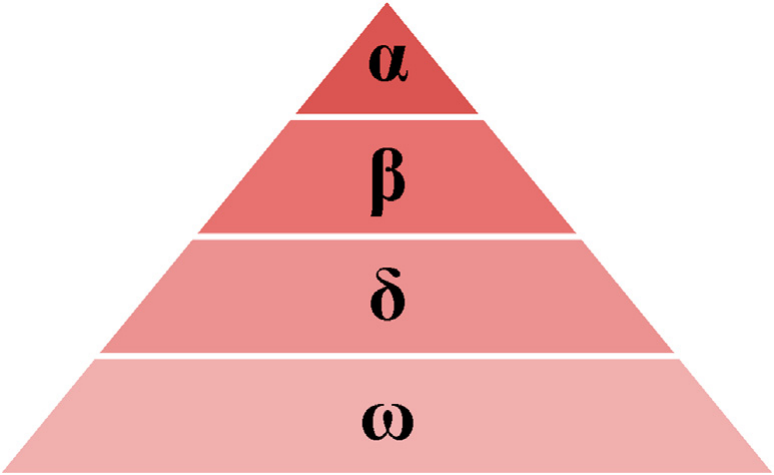
Leadership hierarchy of grey wolves.

In 2014, MIRJALILI et al. [16, 17], developed the grey wolf optimization algorithm which emulates the hierarchy of leadership in wolves known as group hunting. The grey wolf is a part of the CANIDAE family. This family often prefers to live within a group and is characterized by strict social hierarchy. To emulate the leadership hierarchy of grey wolves, four groups α، β، δ، and ω are defined as shown in Figure 3.

The leader could be a male or female and is named as α that is often responsible for making the decisions. The dominant wolf orders must be followed by the group where the group of wolves β is subordinate wolves that help in decision-making. In addition, wolf β is one of α advisers in the group, while the grey wolf ω is the wolf with lowest position in the hierarchy of leadership. If the wolf is not any of α, β, ω, then it is called δ that dominates ω and sends reports to α and β. The mathematical modeling of hunting techniques and the societal ladder of wolves was figured out in order to develop and implement GWO algorithm. The algorithm was tested using standard test functions and results showed that this algorithm overpassed artificial intelligence techniques. Furthermore, the algorithm was successfully applied to solve various engineering optimization problems. Most Swarm techniques used to solve optimization problems cannot have a leader during the entire processing period. This problem is usually solved in GWO where grey wolves have a specific hierarchical social sequence. Moreover, GWO algorithm easy to implement as it contains only some parameters which makes it superior to its predecessors [18, 19, 20, 21, 22].

The GWO algorithm steps can be summarized as follows:

✓ Initialization of GWO algorithm parameters such as search agents (G_S_), size of design variables (Gd), vectors α, A, C and the maximum number of repetition Iter_max_.

The main three steps of hunting that have been emulated using this algorithm are: searching for prey, surrounding it, and attacking it. This algorithm also requires setting the following parameters:

❖ Alpha, Beta, Delta initialization.
❖ Number of search agents.
❖ Maximum number of iteration.
❖ Number of locations selected for searching around the main visited locations.
❖ Stop criterion.

**Figure 4:**
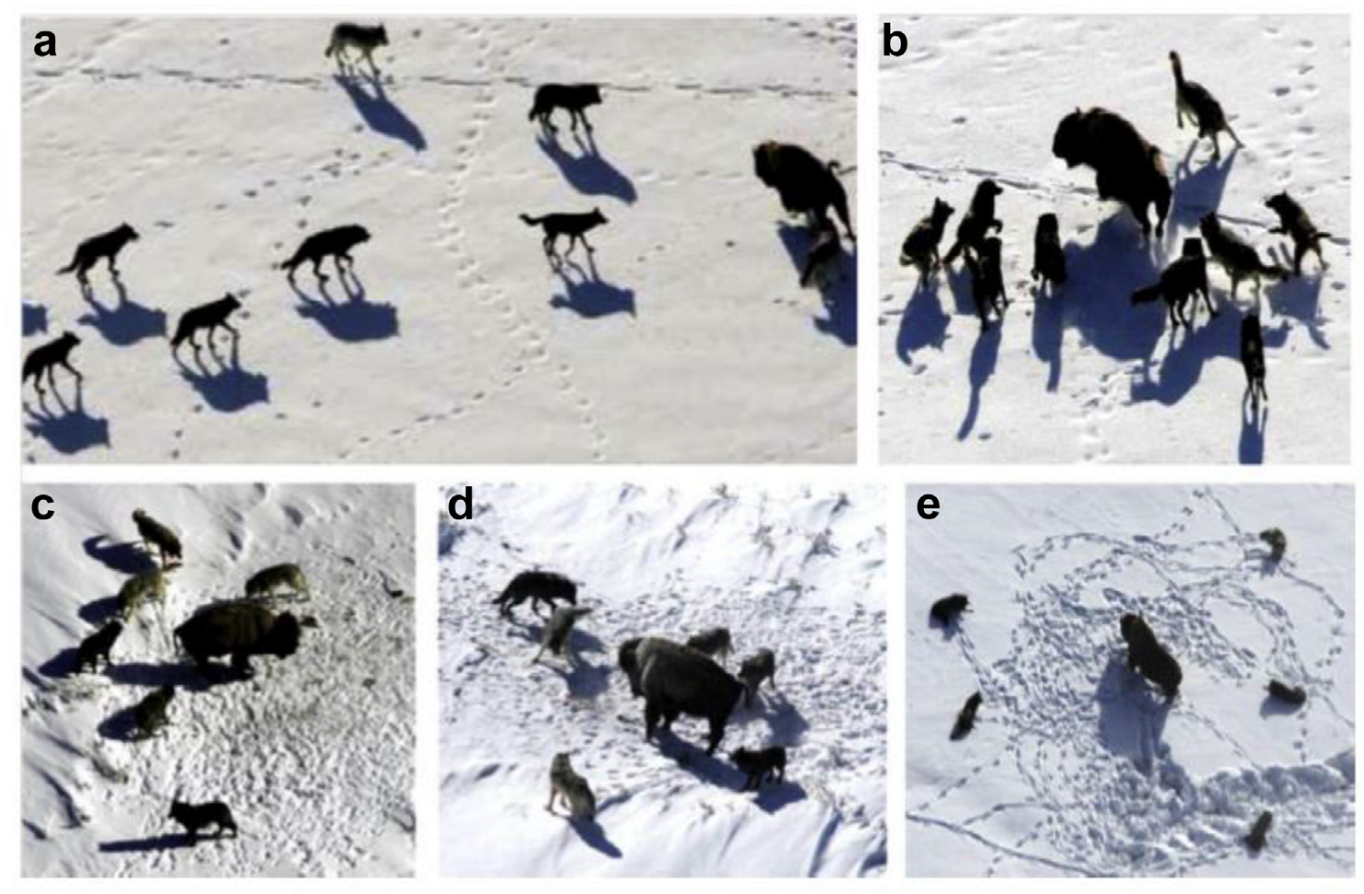
Hunting behavior by grey wolves (a) Tracking, approaching, observing prey, (b-c-d) Chasing prey, surrounding them and harassing them until they stop moving, (e) Attacking prey.

**Figure 5:**
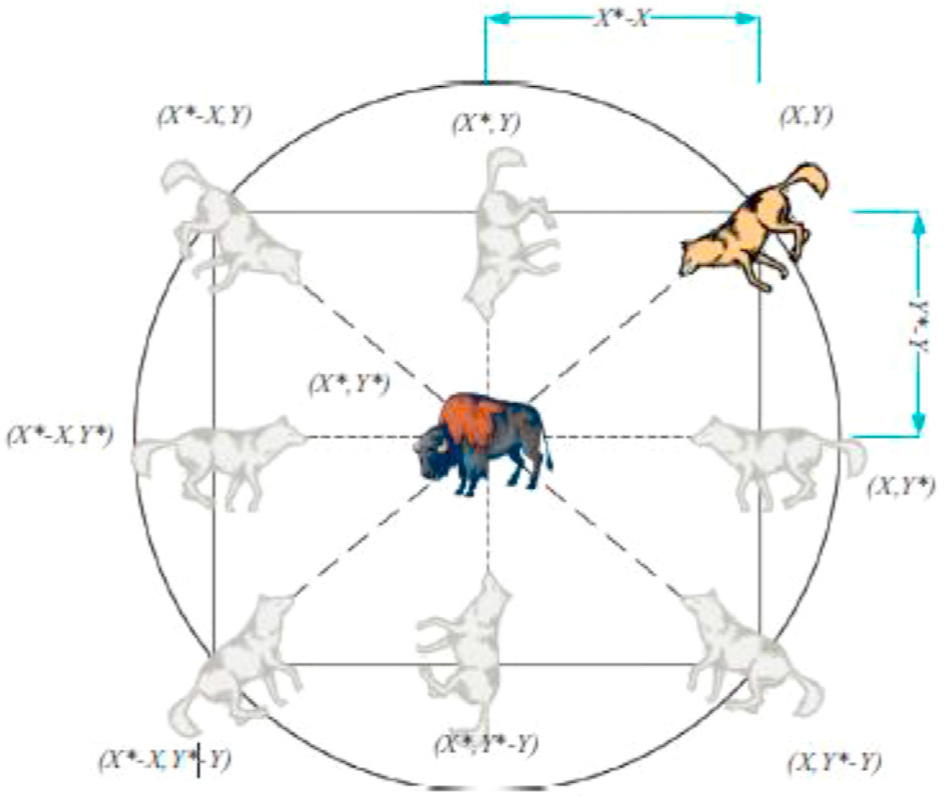
Two-dimensional location vectors and their possible upcoming locations.

**Figure 6:**
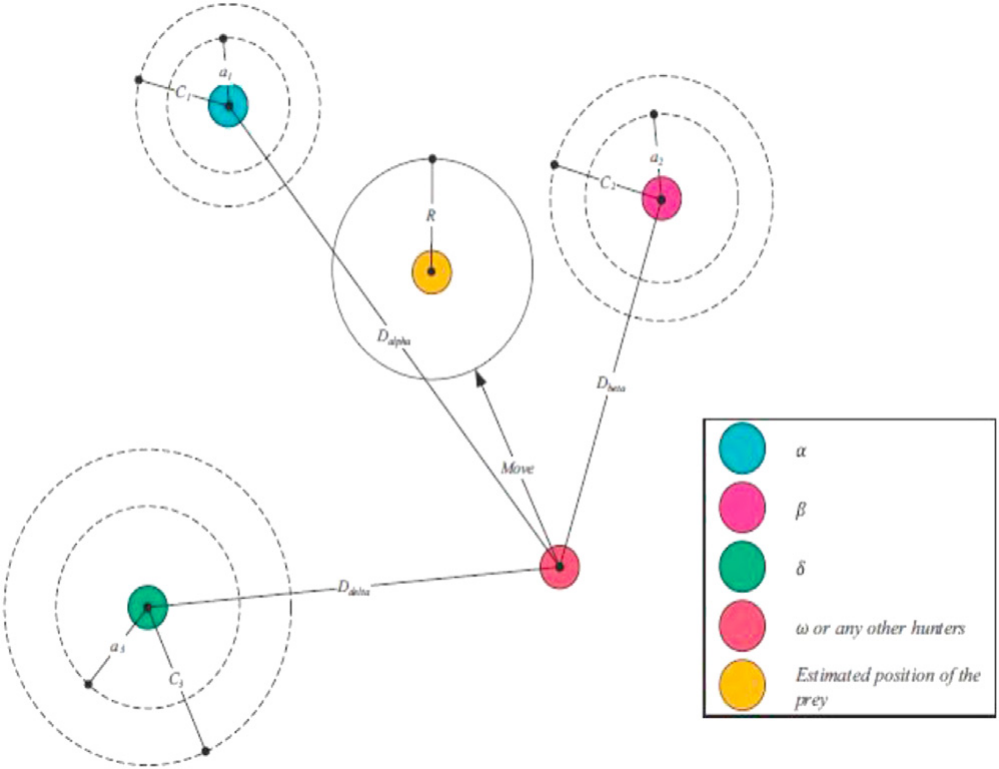
Locations updates in GWO.

**Figure 7:**
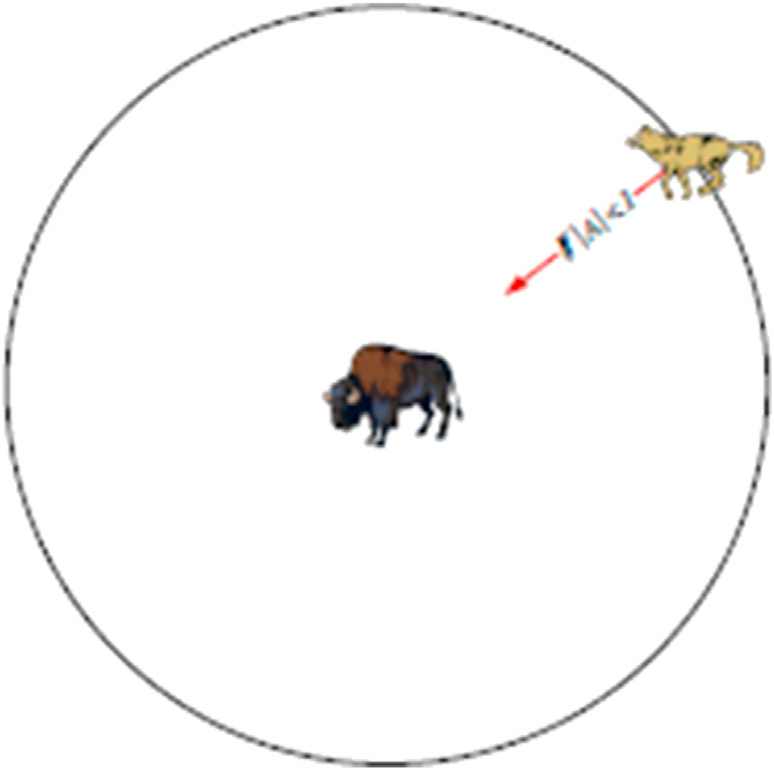
Attacking prey.

The main steps for grey wolf hunting are illustrated in Figures 4, 5, 6, and 7.

❖ Tracking, approaching, and observing prey.
❖ Chasing prey, surrounding them and harassing them until they stop moving.
❖ Attacking prey.

In order to model the social hierarchy of wolves and obtain the gray wolf model, it is assumed that the best solution is α, then β, then δ, and then the rest of solutions ω are considered as candidate solutions.

✓ Generating wolves randomly depending on the size of the group (number of wolves in the group), and these wolves can be expressed mathematically according using the following equations:

(1) $$Wolves=\left[\begin{array}{ccccc} G_{1}^{1} & G_{2}^{1} & \begin{array}{cc} G_{3}^{1} & \begin{array}{cc} . & . \end{array} \end{array} & G_{G_{d}-1}^{1} & G_{G_{d}}^{1}\\ G_{1}^{2} & G_{2}^{2} & \begin{array}{ccc} G_{3}^{2} & . & . \end{array} & G_{G_{d}-1}^{2} & G_{G_{d}}^{2}\\ . & . & \begin{array}{ccc} . & . & \begin{array}{cc} . & \end{array}. \end{array} & . & .\\ . & . & \begin{array}{ccc} . & \begin{array}{cc} . & . \end{array} & . \end{array} & . & .\\ G_{1}^{G_{S}} & G_{2}^{G_{S}} & \begin{array}{ccc} G_{3}^{G_{S}} & . & . \end{array} & G_{G_{d}-1}^{G_{S}} & G_{G_{d}}^{G_{S}} \end{array}\right]$$

Where $G_{i}^{J}$ is the initial value of group J for wolves i.

✓ Assessment the fitness value of each hunting agent:

Wolves x Follow these three wolves to model the encirclement behavior, which can be expressed by the following equations:

(2) $$\vec{D}=\left|\vec{C}.{\vec{X}}_{p}\left(t\right)-\vec{X}\left(t\right)\right|$$

(3) $$\vec{X}\left(t+1\right)={\vec{X}}_{p}\left(t\right)-\vec{A}.\vec{D}$$

Where T is the current iteration.

$\vec{A},\vec{C}$ Are coefficient vectors.

${\vec{X}}_{p}$ Prey location vector.

$\vec{X}$ Grey wolves location vector.

Vectors$\vec{A},\vec{C}$ are given according to the following equations [9]:

(4) $$\vec{A}=2\vec{a}.{\vec{r}}_{1}-\vec{a}$$

(5) $$\vec{C}=2.{\vec{r}}_{2}$$

Where $\vec{a}$ components decrease linearly from 2→0 throughout the iteration and vectors ${\vec{r}}_{1},{\vec{r}}_{2}$are random vectors ranges within [0,1].

✓ determination of best hunting agent [9]:

(6) $$D_{\alpha }=\left|{\vec{C}}_{1}.{\vec{X}}_{\alpha }-\vec{X}\right|$$

(7) $$D_{\beta }=\left|{\vec{C}}_{2}.{\vec{X}}_{\beta }-\vec{X}\right|$$

(8) $$D_{\delta }=\left|{\vec{C}}_{3}.{\vec{X}}_{\delta }-\vec{X}\right|$$

(9) $${\vec{X}}_{1}={\vec{X}}_{\alpha }-{\vec{A}}_{1}.\left({\vec{D}}_{\alpha }\right)$$

(10) $${\vec{X}}_{2}={\vec{X}}_{\beta }-{\vec{A}}_{1}.\left({\vec{D}}_{\beta }\right)$$

(11) $${\vec{X}}_{3}={\vec{X}}_{\delta }-{\vec{A}}_{1}.\left({\vec{D}}_{\delta }\right)$$

✓ Determination of the current location for hunting agent using the following equation [20, 21, 22, 23, 24, 25]:

(12) $$\vec{X}\left(t+1\right)=\frac{{\vec{X}}_{1}+{\vec{X}}_{2}+{\vec{X}}_{3}}{3}$$

✓ Assessment of fitness value for each stalker.
✓ Updating the values ${\vec{X}}_{\alpha },{\vec{X}}_{\beta },{\vec{X}}_{\delta }$.
✓ Testing the stop criterion:
  • If the iteration Iter reaches its maximum value Iter_max_ then the best value must be shown as a result of searching.
  • If Iter does not reach Iter_max_, then step 5 is repeated.

**Figure 8:**
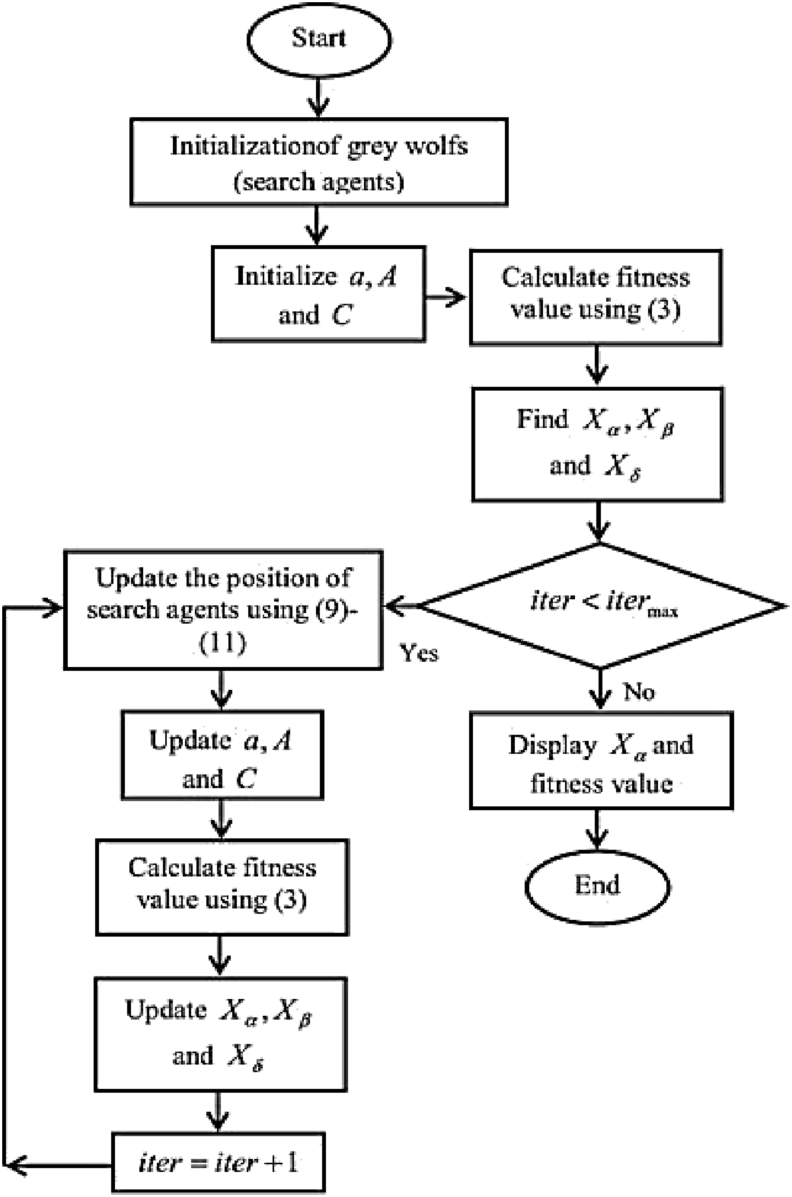
The flowchart of GWO algorithm.

The final location may be in a random spot within a certain domain that can be identified by locations of α, β, δ in the search space. In other words, wolves α, β, δ determine the location of the prey while the rest of the wolves update their locations randomly around this prey. Figure 8 shows the GWO grey wolf optimization flowchart.

The grey wolf algorithm can be Characterized by the following:

❖ Ease of implementation.
❖ Less storage memory is required.
❖ Fast convergence due to the continuous decrease of the search space and the fewer number of decision-making variables in comparison with other algorithms.

## 3 Studied drive system

**Figure 9:**
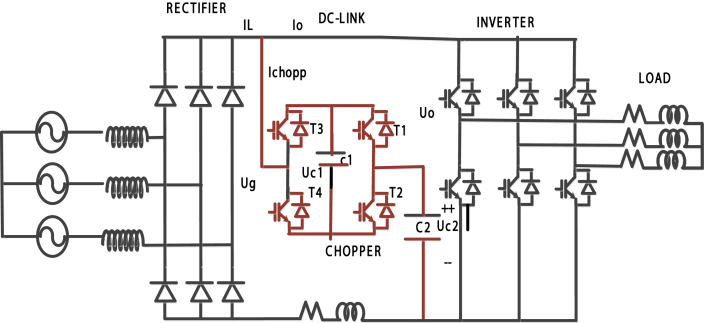
Studied system.

**Figure 10:**
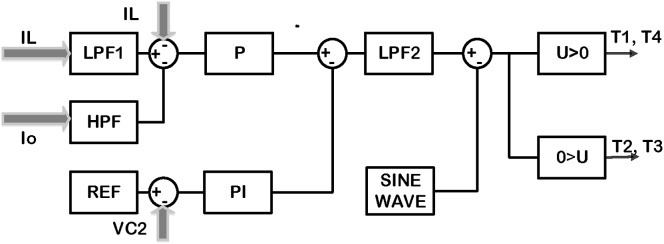
Control plan used to generate the voltages biases necessary for transistors chopper operations of the studied drive system.

The studied system shown in Figure 9 is a VSDS that comprises an electric grid, a rectifier, and a DC-link integrated with a four-quadrant chopper in order to enhance the power quality of the system. For the ease of the study, the inverter with the motor has been substitute by an ohmic load. The average load current in DC-link does not pass through the active components of the circuit in the studied system. Four-quadrant chopper acts like a hybrid (serial-parallel) filter that allows only the high-frequency currents to flow. In order to regulate the bidirectional current flow, four electronic switches have been used. The control scheme used for chopper transistors is shown in Figure 10. It consists of Low pass filter, high pass filter, proportional - integral PI controller, proportional - integral - differential control with the filter PIDN controller. The control signals are obtained by comparing the sinusoidal signal with the control signal [[26], [27]].

The Low Pass Filter LPF1 is used to smoothen the high-frequency parts in load currents. PID and PIDN controllers are used to regulate DC-link current and voltage respectively. The High Pass Filter HPF detects high-frequency components and bans the low-frequency components of the dc-link current. The Low Pass Filter LPF2 reduces the frequency switching ripples.

**Figure 11:**
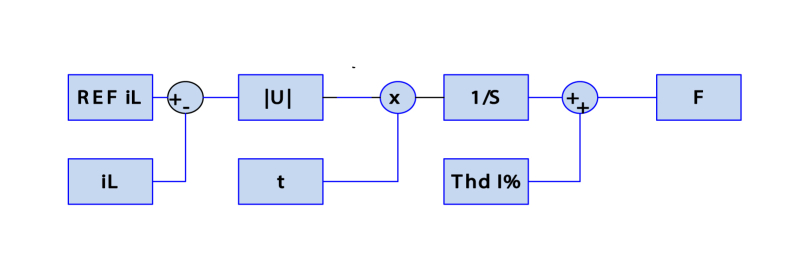
Detailed schematic diagram of the objective function.

Objective function: The objective function is the integration of the absolute value of the difference between the optimal value and real value of load current multiplied by time summed with the value of total harmonic distortion factor THD% of the input current. Figure 11 shows a detailed schematic diagram of the objective function. The objective function is defined by the following equation:

(13) F=ITAE=Thd(I)%+∫0∞|IL−Ref(IL)|.t.dt

### 3.1 GWO algorithm parameters

In order for GWO algorithm to adjust PID and PIDN controllers' parameters, the algorithm has been written in MATLAB as a MALAB script and this script has been integrated with the MATLAB model of the studied drive system. The initial parameters of GWO algorithm parameters have been set as follows:

**Table:** 

Number of hunting agents:	20
Number of unknown variables:	6
Maximum number of iteration:	100

While the PSO algorithm parameters were set as:

**Table:** 

Swarm size	50
Maximum number of iteration	150
C1 = C2	2
W_max_	0.9
W_min_	0.2

The maximum and minimum limits of variables were selected as shown in Tables 1 and 2.

**Table 1:** The minimum and maximum limits of PIDN controller used to control the four-quadrant chopper transistors.

PIDN controller	Minimum limit	Maximum limit
proportional gain K_P_	20	40
integral gain K_I_	0	10
Differential gain K_D_	0	10
N filter	1	500

**Table 2:** The minimum and maximum limits of PI controller used to control the four-quadrant chopper transistors.

PI controller	Minimum limit	Maximum limit
proportional gain K_P_	0	100
integral gain K_I_	0	10

## 4 Results and discussion

After running the proposed algorithms MATLAB scripts, the best parameters corresponding to the optimal solution (the minimum value of the objective function) were as shown in Tables 3 and 4.

**Table 3:** The optimal values of PIDN controller parameters obtained using the grey wolf optimization algorithm.

PIDN controller				
Gain	N	K_D_	K_I_	K_P_
GWO	1.01952	0.0514288	0.0441717	20
PSO	8.0256	2.2354	6.37125	29.1589

**Table 4:** The optimal values of PI controller parameters obtained using the grey wolf optimization algorithm.

PI controller		
Gain	K_I_	K_P_
GWO	3.94672	0.006
PSO	6.2354	0.877

**Figure 12:**
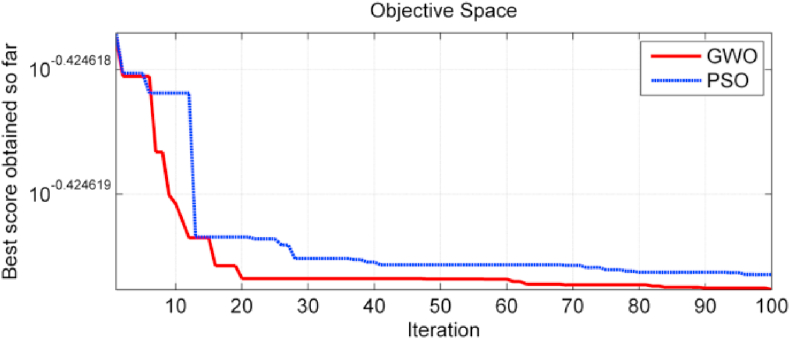
The values of the objective function when using GWO and PSO algorithms.

The minimum value reached by objective function is (10^−0.424618^). GWO algorithm has reached this value in about 90 iterations, while PSO algorithm has needed 95 iterations to reach the minimum value of objective function. Figure 12 shows the superiority of the GWO when compared with PSO in terms of reaching to the minimum value of objective function.

**Figure 13:**
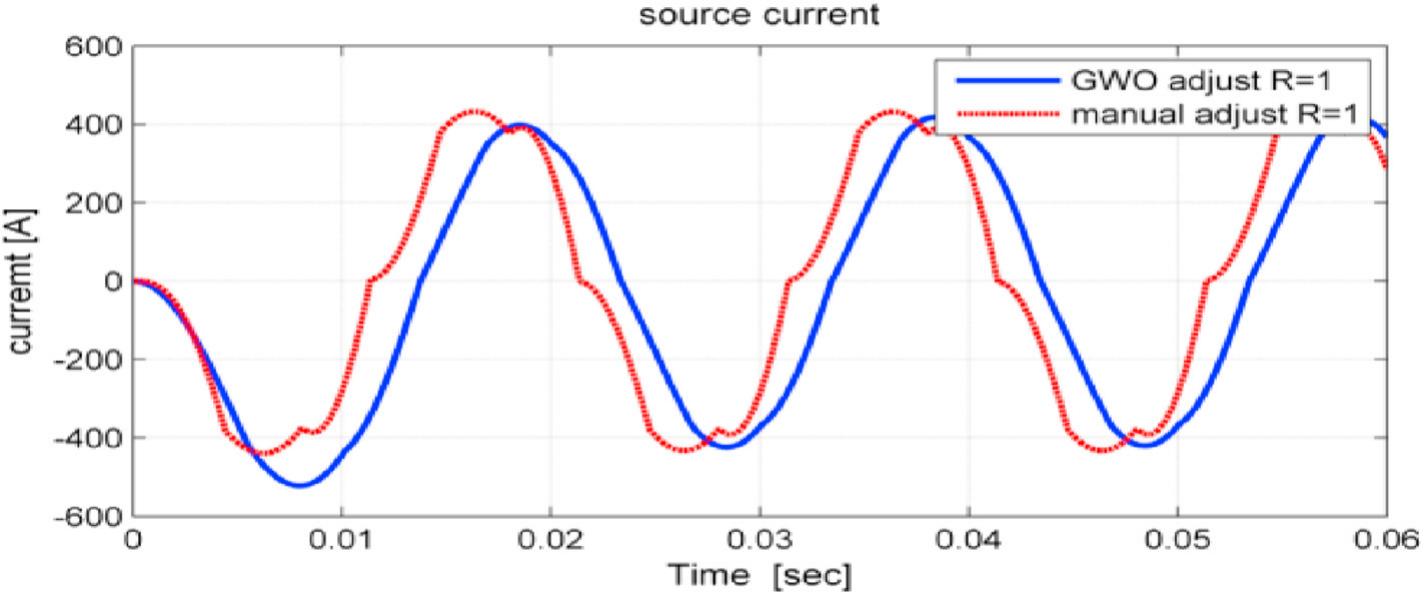
System input currents when using both PSO and GWO for controllers' parameters tuning.

**Figure 14:**
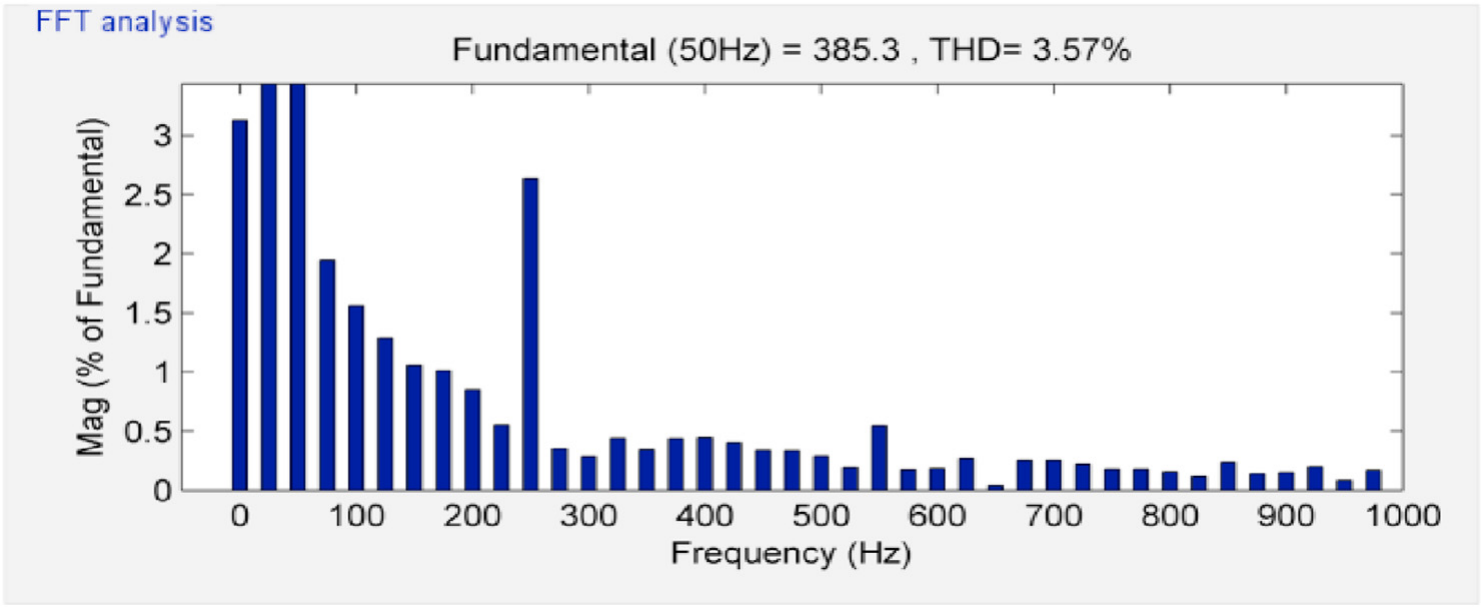
The total harmonic distortion factor of the studied system input current when using GWO.

Figure 13 shows the input current of the VSDS when parameter values of the PIDN and PI are adjusted using the GWO and PSO that has been denoted by manual adjust. It is observed that current wave became smoother and input current THD% decreases from 9.76% when using PSO to 3.57% when using GWO as shown in Figure 14.

**Figure 15:**
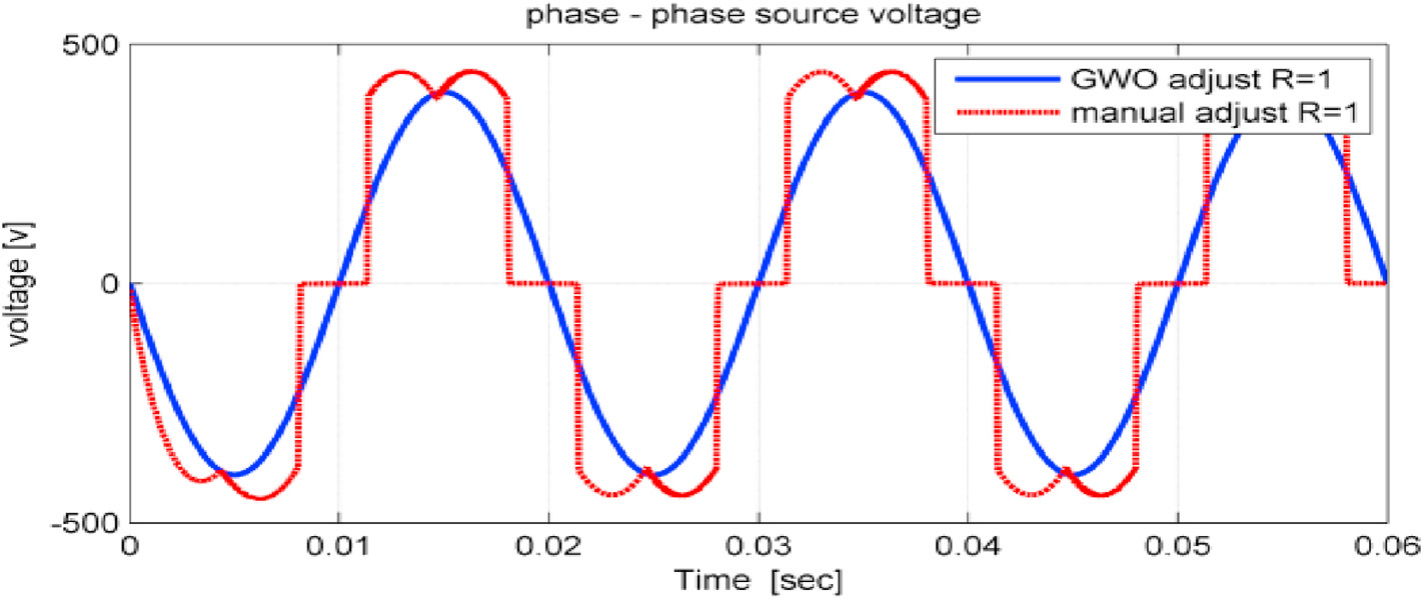
Phase to phase input voltage using GWO and PSO.

The phase to phase input voltage of the studied system was greatly improved after adjusting the controller's parameters using GWO algorithm, as shown in Figure 15. It is noticed that the voltage ripples were eliminated.

**Figure 16:**
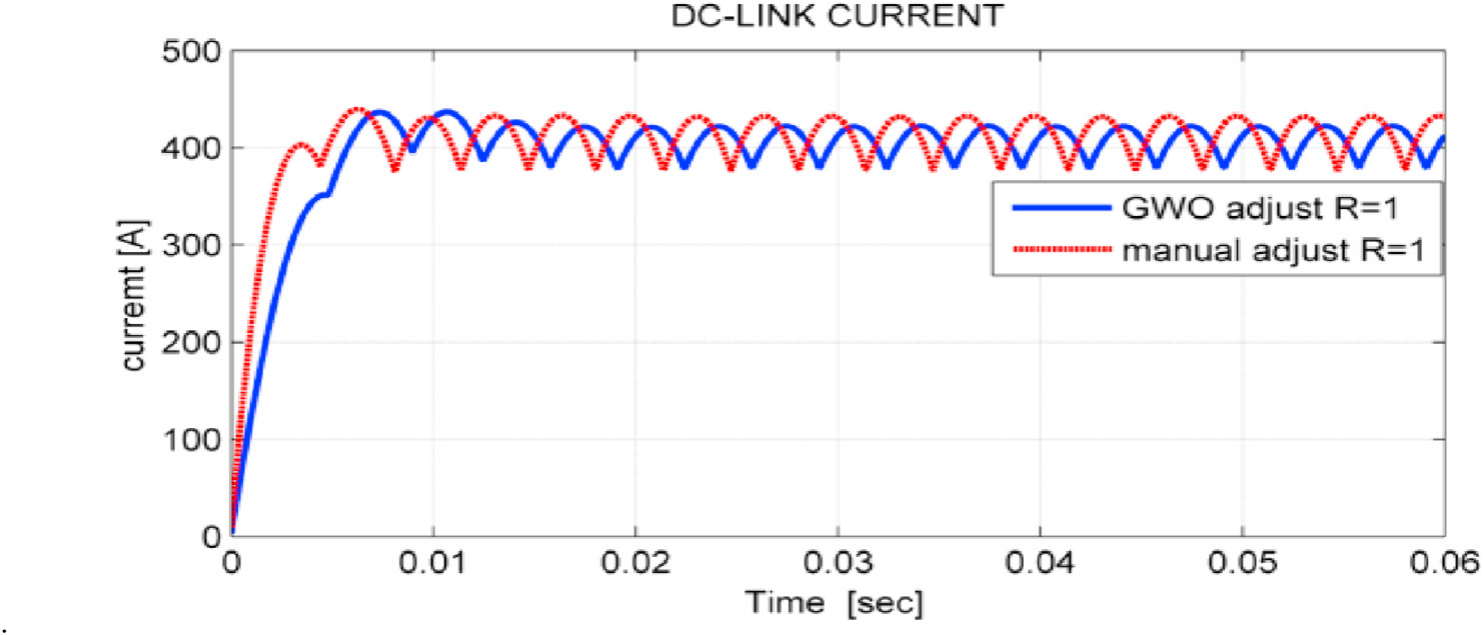
Dc-link current of the studied system using GWO and PSO algorithms.

The ripple factor of DC- link current is reduced from 0.04 using PSO to 0.03 using GWO. Figure 16 shows the DC-link current of the studied system in both cases.

**Figure 17:**
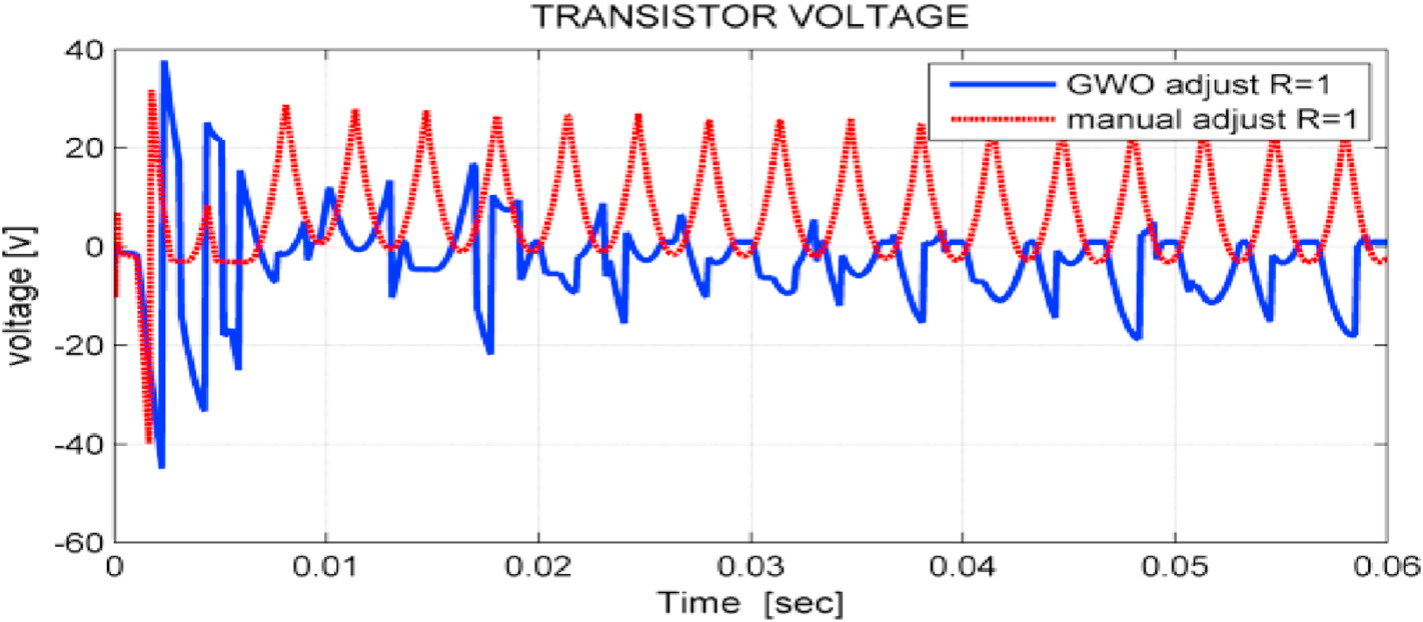
The voltage on chopper transistors in the DC-link of the studied system.

Figure 17 shows voltage drops on the chopper transistors when using GWO and PSO algorithms. It is observed that the voltage drop has been reduced to half when using GWO in comparison with PSO, which reduce the power losses.

When the values of the controller's constants is changed in range of ±10%, the time response of the system is slightly affected. This indicates that the GWO algorithm is effective in reaching the optimal values of the constants in both the dynamic and static working conditions.

When all the parameters of the used controllers are changed within a range of ±10%, the total harmonic distortion factor of the input current becomes about 4.09% and the DC-link ripple factor is about 0.034 which confirms the algorithm's ability to operate in different environments.

The comparison between the values of the system specifications and characteristics when adjusting the controller parameters using different techniques are shown in Table 5.

**Table 5:** Comparison between the values of power quality specifications when adjusting the controller parameters using different algorithms.

Algorithm	Fuzzy Logic	GA	PSO	GWO
Values				
THD_I_	12%	10.2%	9.76%	3.57%
THD_V_	5%	5%	4.8%	2%
Voltage RF	0.13	0.12	0.04	0.03

## 5 Conclusions and recommendations

Grey wolf optimization (GWO) and particle swarm optimization (PSO) algorithms have been used in this paper to adjust parameters of controllers PI, PIDN. These controllers are used in SPWM circuit that controls chopper's switches. GWO algorithm has improved the power quality specifications of the studied system by reducing both the total harmonic distortion factor THD% of the system input currents and the ripple factor RF of the DC-link. GWO need less number of iterations to reach the optimal solution when compared with PSO in both dynamic and static operation conditions of the system. We recommend using other optimization algorithms such as flower pollination algorithm FPA and imperialist competitive algorithm ICA to achieve the best time response of the studied system.

## Declarations

### Author contribution statement

Safwan Nadweh, Ola Khaddam, Ghassan Hayek, Bassan Atieh & Hassan Haes Alhelou: Conceived and designed the experiments; Performed the experiments; Analyzed and interpreted the data; Wrote the paper.

### Funding statement

This research did not receive any specific grant from funding agencies in the public, commercial, or not-for-profit sectors.

### Competing interest statement

The authors declare no conflict of interest.

### Additional information

No additional information is available for this paper.
